# Single nucleotide polymorphisms in the bovine MHC region of Japanese Black cattle are associated with bovine leukemia virus proviral load

**DOI:** 10.1186/s12977-017-0348-3

**Published:** 2017-04-04

**Authors:** Shin-nosuke Takeshima, Shinji Sasaki, Polat Meripet, Yoshikazu Sugimoto, Yoko Aida

**Affiliations:** 1 Viral Infectious Diseases Unit, RIKEN, 2-1 Hirosawa, Wako, Saitama 351-0198 Japan; 2 Shirakawa Institute of Animal Genetics, Japan Livestock Technology Association, Odakura, Nishigo, Fukushima 961-8061 Japan

**Keywords:** Bovine leukemia virus, Whole genome association study, Major histocompatibility complex

## Abstract

Bovine leukemia virus (BLV) is the causative agent of enzootic bovine leukosis, a malignant B cell lymphoma that has spread worldwide and causes serious problems for the cattle industry. The BLV proviral load, which represents the BLV genome integrated into host genome, is a useful index for estimating disease progression and transmission risk. Here, we conducted a genome-wide association study to identify single nucleotide polymorphisms (SNPs) associated with BLV proviral load in Japanese Black cattle. The study examined 93 cattle with a high proviral load and 266 with a low proviral load. Three SNPs showed a significant association with proviral load. One SNP was detected in the *CNTN3* gene on chromosome 22, and two (which were not in linkage disequilibrium) were detected in the bovine major histocompatibility complex region on chromosome 23. These results suggest that polymorphisms in the major histocompatibility complex region affect proviral load. This is the first report to detect SNPs associated with BLV proviral load in Japanese Black cattle using whole genome association study, and understanding host factors may provide important clues for controlling the spread of BLV in Japanese Black cattle.

**Electronic supplementary material:**

The online version of this article (doi:10.1186/s12977-017-0348-3) contains supplementary material, which is available to authorized users.

## Main text

Bovine leukemia virus (BLV), which infects cattle worldwide [1–6], belongs to the family *Retroviridae* (genus *Deltaretrovirus*), together with human T cell leukemia virus types 1 and 2 (HTLV-1 and -2) [7]. Historically, the economic losses caused by BLV infection were thought to be related only to the onset of bovine leukosis, which occurs in only 1–5% of BLV-infected cows within 5 years post-infection [7]. However, recent reports show that BLV infection also reduces milk production [6, 8–11] and causes a high incidence of infectious disease [12] and reproductive inefficiency, resulting in high culling rates [13]; thus BLV eradication is of utmost importance.

Previous studies show that the proviral load is an important index for estimating the stage of BLV infection because it is associated with disease progression [14–16], lymphocyte count [17], viral biokinetics [18], and virus shedding into saliva and nasal secretions [19]. Indeed, one study shows that cattle with a low proviral load are not a source of BLV transmission [20]. Therefore, determining host factors associated with an increased proviral load is important if we are to develop eradication programs for BLV.

Studies of BLV-associated host factors identified polymorphisms within the bovine major histocompatibility complex (MHC) (BoLA) [21–29]. Recently, Miyasaka et al. revealed that polymorphisms within BoLA class II haplotypes were strongly associated with BLV proviral load in Japanese Black cattle, the main breed of beef cattle in Japan, but less so with that in European breeds [22]. However, no group has undertaken a genome-wide association study (GWAS) to identify such host factors.

**Fig. 1:**
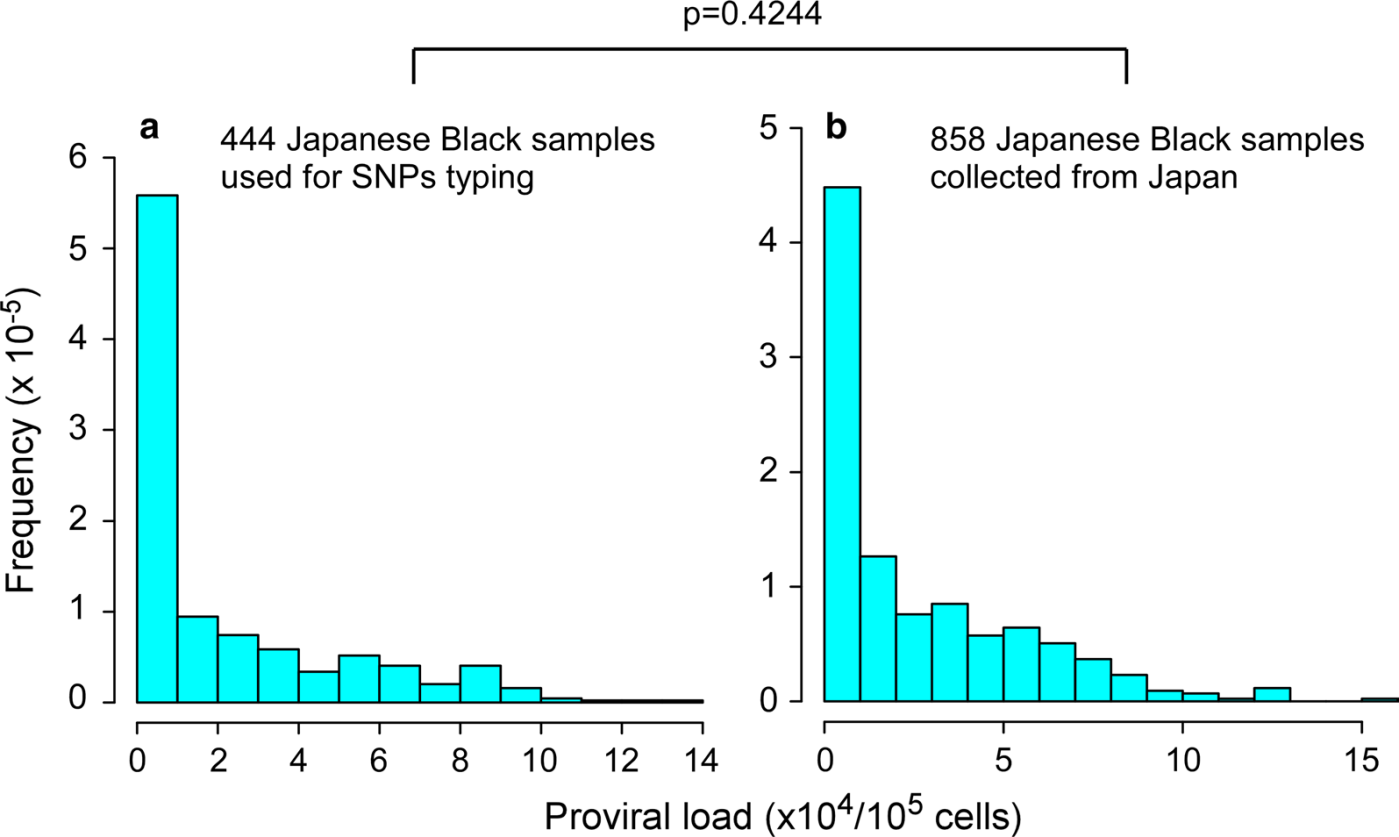
Proviral load estimated from SNP typing of DNA samples from 444 BLV-infected Japanese Black cattle (**a**) and 858 samples from Japanese Black cattle located in 22 prefectures of Japan (**b**) [17]. The proviral load in the 444 test samples was representative of the proviral load in Japanese Black cattle nationwide (*p* value, *p* = 0.4244; F test). Blood (collected in EDTA-2Na) was obtained from 444 Japanese black cows (aged >4 years), and genomic DNA was extracted from whole blood using the QIAsymphony kit (QIAGEN K.K., Tokyo, Japan). The BLV-CoCoMo-qPCR-2 method (RIKEN genesis, Kanagawa, Japan) was used to measure the BLV proviral load in 676 cattle at a single time-point; of these, 444 were positive for BLV and entered into the association study. Briefly, the BLV long terminal repeat region was amplified using a degenerate primer pair (CoCoMo-FRW and CoCoMo-REV) and an FAM-BLV probe. The *BoLA*-*DRA* gene (internal control) was amplified using the primer pair DRA-F and DRA-R and the FAM-DRA probe [31]

Therefore, to identify proviral load-associated polymorphisms, we performed a GWAS using DNA samples from 676 Japanese Black cattle [30]. Genomic DNA was isolated from peripheral blood, and the BLV proviral load was measured using the BLV-CoCoMo-qPCR-2 method [31]. BLV provirus was detected in samples from 444 animals (range, 1 copy/10^5^ cells to 132,230 copies/10^5^ cells; median value, 5498 copies/10^5^ cells) (Fig. 1a). We then compared the proviral load in animals used for the GWAS with that in Japanese Black cattle selected randomly from whole areas of Japan. We found no significant difference in the proviral load between animals used for GWAS and the randomly selected group (Fig. 1b). In most cases, the animals in both groups showed a proviral load of <10,000 copies/10^5^ cells. A proviral load >100,000 copies/10^5^ cells was rare.

**Fig. 2:**
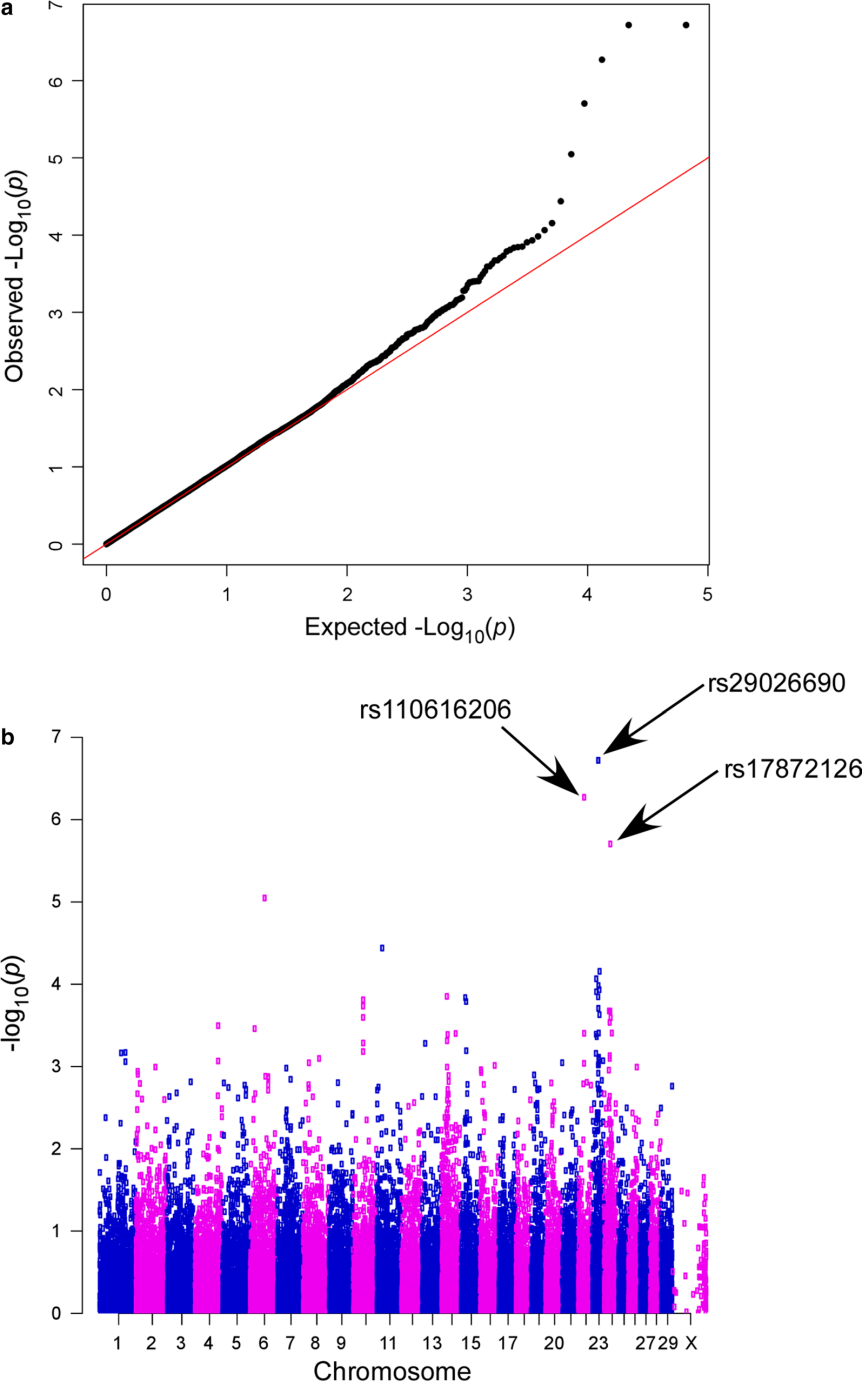
Three-hundred and fifty-nine BLV-infected cows were genotyped using a BovineSNP50 DNA Analysis BeadChip (Illumina Inc., San Diego, CA), and SNPs associated with the BLV proviral load were examined. **a** Quantile–quantile (Q–Q) plot. The observed distribution of the −log_10_ nominal *p* values (*y-axis*) demonstrates a significant departure from the null hypothesis (expected values are shown on the *x-axis*) (λ_GC_ = 1.021). *Red line* represents the line as y = x. **b** Manhattan plot showing the association between 33,006 SNPs (BovineSNP BeadChip) and the BLV proviral load in DNA samples from 359 Japanese Black cattle. The chromosomes are denoted by *different colors* (*blue* odd numbers; *orange* even numbers). The chromosome number is indicated on the *x-axis*. The *blue line* represents the Bonferroni-corrected threshold for genome-wide significance (−log_10_(*p*) = 5.82)

**Fig. 3:**
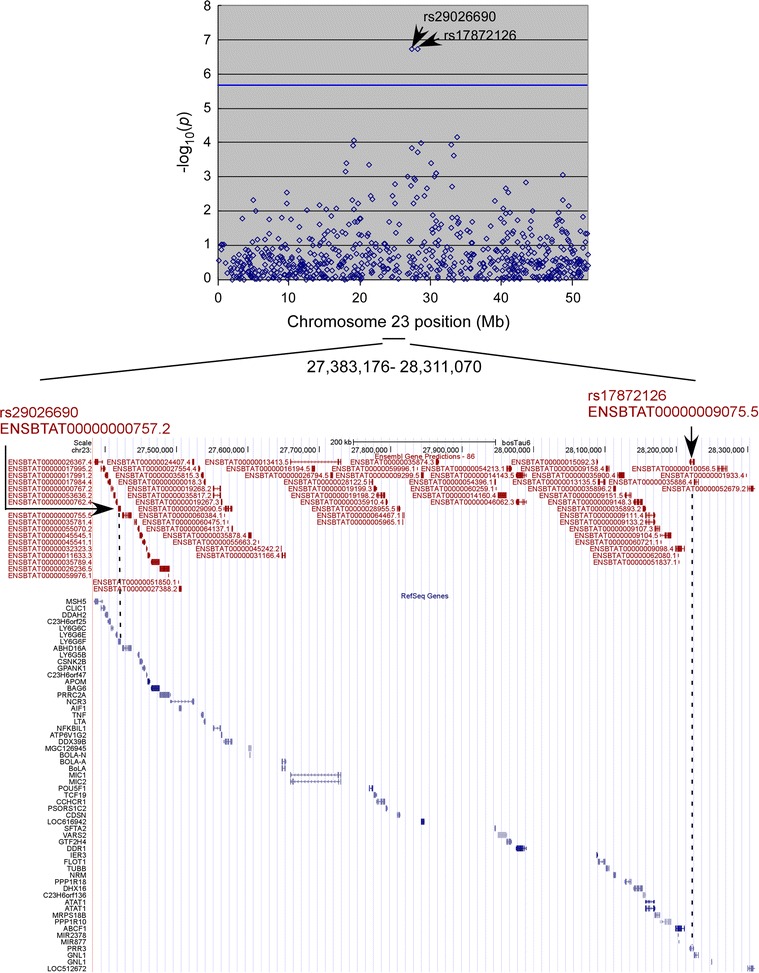
Regional Manhattan plot of the locus on chromosome 23 that harbors SNPs associated with BLV proviral load. The imputed SNPs are shown by *arrows*. Genes (Chr23:27,116,737 to Chr23:28,311,070) are listed, and the positions of SNPs associated with the BLV proviral load are indicated by *arrows*. The *horizontal blue lines* represent the Bonferroni-corrected thresholds for genome-wide significance (−log_10_(*p*) = 5.82). The indicated positions are based on the bovine genome (assembled in UMD3.1)

We categorized the 444 BLV-infected cows into four groups according to proviral load: Low (0 < provirus load ≤ 13,819, 266 heads), Medium (14,237 < provirus load ≤ 40,698, 85 heads), High (42,605 < provirus load ≤ 73,145, 60 heads), and Very High (76,397 < provirus load ≤ 132,230, 33 heads). We then performed a GWAS using these traits as a binary variable, as is done in 93 case (High + Very High group) − 266 control (Low group) studies. The 359 animals were genotyped using a SNP50 K BeadChip comprising probes targeting 54,001 single nucleotide polymorphisms (SNPs). In all, 32,919 autosomal SNPs met the quality control criteria (call rate >99%; minor allele frequency >0.01; Hardy–Weinberg equilibrium, *p* > 0.001). Analyses were then performed using GEMMA software [32], which uses a linear-mixed model approach based on a genetic-relationship matrix estimated from SNP genotypes to model correlations between the phenotypes of sample subjects. The genomic-inflation factor (λ_GC_) for this analysis was 1.021, indicating that a sample was appropriate for inclusion in an association study. The quantile–quantile (Q–Q) plot showed that three SNPs showed a significant deviation from the null hypothesis (Fig. 2b, Bonferroni-corrected threshold for genome-wide significance (*p* < 1.5 × 10^−6^) add threshold line in A). Three significant genome-wide associations were detected: rs29026690 (*p* = 1.91 × 10^−7^, odds ratio = 2.745) and rs17872126 (*p* = 1.91 × 10^−7^, odds ratio = 0.414) on bovine chromosome 23 (BTA23) and rs110616206 (*p* = 5.37 × 10^−7^, odds ratio = 6.589) on BTA22 (Fig. 2b; Table 1). The two SNPs on BTA23 were found within an 800 Kb window located at 27,421,348–28,223,274 bp; these two SNPs did not show linkage disequilibrium (LD) (*r* ^*2*^ = 0.117), indicating that BTA23 harbored two independent quantitative trait loci (QTL)s (Figs. 2b, 3; Table 1).

**Table 1:** SNPs showing a significant association with BLV proviral load

Chromosome	Ilumina_ID^a^	Reference cluster ID^b^	Position^c^	*p*	Minor allele^d^	Minor allele^d^ (case)	Minor allele^d^ (control)	Major allele^e^	Odds ratio^f^
23	Hapmap57616-rs29026690	rs29026690	27421348	1.91 × 10^−7^	A	0.2903	0.1297	G	2.745
23	ARS-BFGL-NGS-113235	rs17872126	28223274	1.91 × 10^−7^	G	0.2849	0.4906	A	0.414
22	Hapmap33580-BTA-136506	rs110616206	27280154	5.37 × 10^−7^	A	0.0914	0.01504	G	6.589

^a^SNP ID assigned by Illumina, Inc

^b^Reference SNP (refSNP) ID assigned in the single nucleotide polymorphism database (dbSNP)

^c^Positions are based on the bovine genome, assembled in UMD3.1

^d^Minor allele is minor frequency allele determined in this study

^e^Major allele is major frequency allele determined in this study

^f^Odds ratio is the effective value for estimating how strongly the SNPs associated to the proviral load, using following formula

$$ {\text{Odds}}\;{\text{ratio}} = \frac{{\frac{{Minor\,allele\;frequency\,\left( {case} \right)}}{{Minor\;allele\;frequency\;\left( {control} \right)}}}}{{\frac{{Major\;allele\;frequency\;\left( {case} \right)}}{{Major\;allele\;frequency\;\left( {control} \right)}}}} $$

Genes within or near these regions were then analyzed using the UMD3.1 genome assembly tool. Hapmap57616-rs29026690 (27,421,348 bp on BTA23) was located between ENSBTAG00000000580 and ABHD16A (ENSBTAG00000000578) (Additional file 1: Table S1), whereas ARS-BFGL-NGS-113235 (28,223,274 bp on BTA23) was located between the 4th and 5th exons of *PRR3* (ENSBTAG00000006914). These SNPs reside within the BoLA class III and class I regions, respectively (Figs. 2b, 3; Additional file 1: Table S1). Therefore, the gene density was much higher than that in other areas of the genome, and a number of candidate genes that could be used to estimate proviral load were present around the detected SNPs [33]. Hapmap33580-BTA-136506 was located on the centromeric side of BTA22, at a distance of 6.5 kb from the *CONTACTIN3 (CNTN3)* gene (Table 1; Additional file 1: Table S1, Additional file 2: Fig. S1).

To the best of our knowledge, this is the first report to detect SNPs associated with BLV proviral load in Japanese Black cattle using GWAS. Two of the identified SNPs were located in the BoLA region. We found it interesting that these two SNPs were located within the class III and class I regions because a previous study reported involvement of only class II genes [22]. The genome reference sequences for the BoLA region have many gaps, mainly because class I genes were difficult to genotype, making associations with class I genes difficult to determine. Target resequencing of high density SNPs across the MHC region using a next generation sequencer should be undertaken to confirm which genes are truly responsible for regulating the proviral load. Our result showed that the MHC polymorphism is important factor for proviral load. The reason why MHC polymorphisms were associated with proviral load is the polymorphism of classical MHC directly associate with antigen presentation and the difference of antigen presentation in each allele leads to the immunological difference in each host.

Taken together, the results described herein show that MHC genotyping of class III and class I alleles can identify cows with a low proviral load. In the farm with high infection rate, eliminating high proviral load cow is an effective way for eradicating BLV because proviral load is major risk factor for transmitting BLV to other host [20]. Therefore, farmer should frequently check the proviral load because the proviral load is variable, although it is not cost-effective. Taken together with the information of our finding 3 SNPs and our previously report about resistant BoLA class II allele [22], we can identify the BLV resistant cow. It will be helpful to develop a low cost method of eradicating BLV from farms because we can reduce the frequently measurement of proviral load.

## Additional files

**Additional file 1: Table S1.** Genes around rs110616206 SNP (Tab "CNTN3") and rs29026690 and rs17872126 SNPs (Tab "BTA23").

**Additional file 2: Figure S1.** Regional Manhattan plot showing the association of 33,006 SNPs (BovineSNP BeadChip) with BLV proviral load in 359 Japanese Black cattle. Regional plot of the locus on chromosome 22 that harbors SNPs associated with BLV proviral load. The imputed SNPs are indicated by arrows. The horizontal blue lines represent the Bonferroni-corrected thresholds for genome-wide significance (−log_10_(*p*) = 5.82). The indicated positions are based on the bovine genome (assembled in UMD3.1).
