# Supplementary figures and images for: Single nucleotide polymorphisms in the bovine MHC region of Japanese Black cattle are associated with bovine leukemia virus proviral load
Source: Retrovirology. 2017 Apr 4;14:24. doi: 10.1186/s12977-017-0348-3 (PMC5379713; doi:10.1186/s12977-017-0348-3)

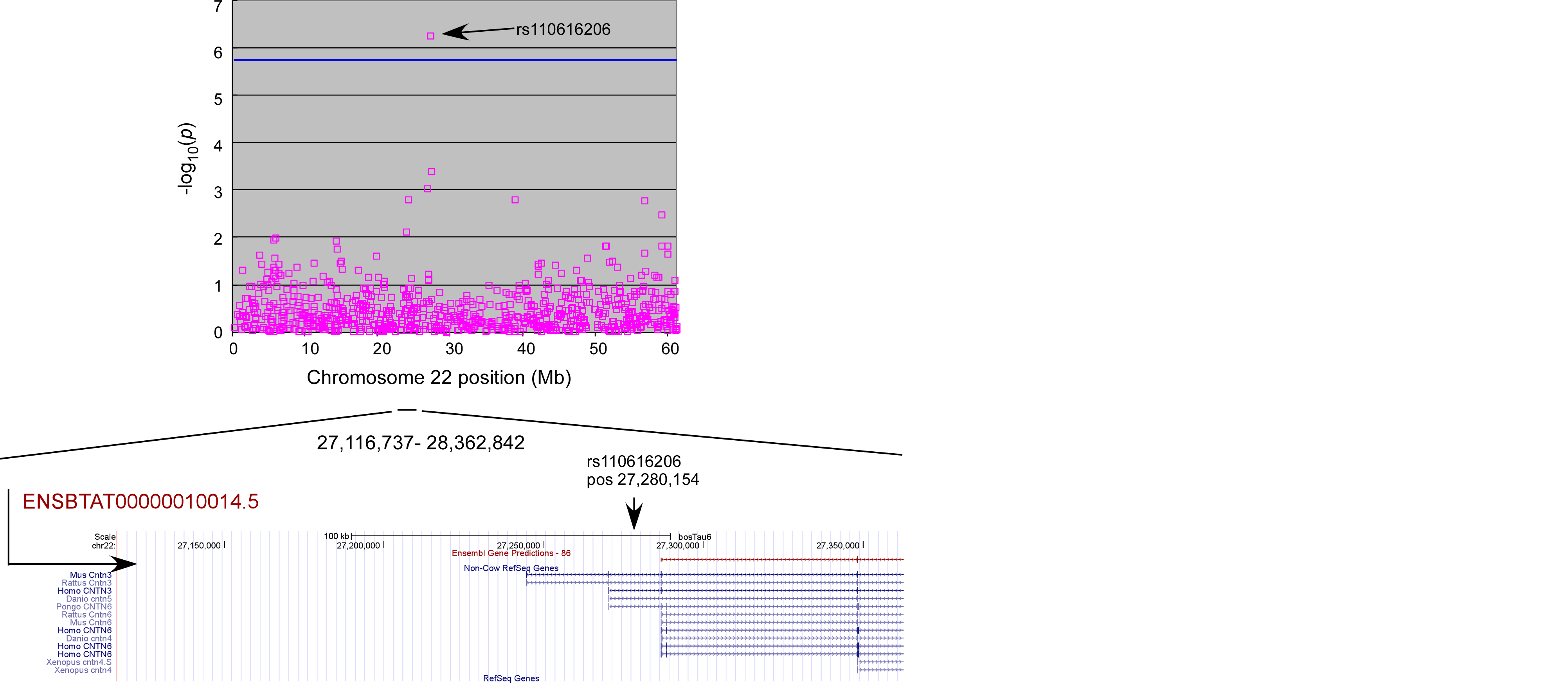

Supplement: Supplementary file 2 — Additional file 2: Figure S1. Regional Manhattan plot showing the association of 33,006 SNPs (BovineSNP BeadChip) with BLV proviral load in 359 Japanese Black cattle. Regional plot of the locus on chromosome 22 that harbors SNPs associated with BLV proviral load. The imputed SNPs are indicated by arrows. The horizontal blue lines represent the Bonferroni-corrected thresholds for genome-wide significance (−log10(p) = 5.82). The indicated positions are based on the bovine genome (assembled in UMD3.1). [file 12977_2017_348_MOESM2_ESM.tif]
